# Using noise to distinguish between system and observer effects in multimodal neuroimaging

**DOI:** 10.3389/fncom.2025.1693279

**Published:** 2025-10-17

**Authors:** Erik D. Fagerholm, Hirokazu Tanaka, Gregory Scott, Robert Leech, Federico E. Turkheimer, Peter Zeidman, Karl J. Friston, Milan Brázdil

**Affiliations:** ^1^First Department of Neurology, St. Anne’s University Hospital and Faculty of Medicine, Masaryk University, Brno, Czechia; ^2^Faculty of Information Technology, Tokyo City University, Tokyo, Japan; ^3^Department of Brain Sciences, Imperial College London, London, United Kingdom; ^4^Department of Neuroimaging, Institute of Psychiatry, Psychology & Neuroscience, King’s College London, London, United Kingdom; ^5^Functional Imaging Laboratory, Department of Imaging Neuroscience, Queen Square Institute of Neurology, University College London, London, United Kingdom

**Keywords:** multimodal neuroimaging, cross-scale integration, generative modeling, observer-system disambiguation, high-frequency broadband activity, stochastic differential equations, Bayesian model comparison

## Abstract

**Introduction:**

It has become increasingly common to record brain activity simultaneously at more than one spatiotemporal scale. Here, we address a central question raised by such cross-scale datasets: do they reflect the same underlying dynamics observed in different ways, or different dynamics observed in the same way? In other words, to what extent can variation between modalities be attributed to system-level versus observer-level effects? System-level effects reflect genuine differences in neural dynamics at the resolution sampled by each device. Observer-level effects, by contrast, reflect artefactual differences introduced by the nonlinear transformations each device imposes on the signal. We demonstrate that noise, when incorporated into generative models, can help disentangle these two sources of variation.

**Methods:**

We apply this noise-based approach to simultaneously recorded high-frequency broadband signals from macroelectrodes and microwires in the human hippocampus.

**Results:**

Most subjects show a complex mixture of system- and observer-level contributions to their time series. However, in one subject, the cross-scale difference is statistically attributable to an observer-level effect—i.e., consistent with the same dynamics at both microwire and macroelectrode scales.

**Discussion:**

This study shows that noise can be used in empirical datasets to determine whether cross-scale variation arises from differences in neural dynamics or differences in observer functions.

## 1 Introduction

Patterns of electrical activity propagate through networks of the brain over a wide range of spatial and temporal scales (Engel et al., 2021). This activity can be recorded by different types of neuroimaging devices, each of which captures electrical signals at a specific resolution–much like a microscope only reveals one level of magnification, depending on the properties of its lens. Electrophysiological experiments increasingly take advantage of this by combining recordings from microwires and macroelectrodes–which differ in spatial sampling and sensitivity (Keles et al., 2024)–and analyzing their high-frequency broadband (HFB) signals. In this study, we refer to micro-HFB and macro-HFB as the power extracted from microwire and macroelectrode recordings, which approximately correspond to local field potentials (LFP) and intracranial EEG (iEEG), respectively.

When signals from micro- and macro-HFB are recorded simultaneously, they offer complementary views of neural dynamics across scales, raising the question of how to compare or combine the information they provide. However, cross-scale measurements are complicated by the fact that every imaging device invariably distorts the brain’s electrical signals in unique and often nonlinear ways (Frank et al., 2024). These device-specific distortions introduce an ambiguity as to whether the variation between multimodal signals arises from differences across scales in the brain, or from differences in the properties of the devices. The challenge of addressing this ambiguity can be framed in terms of a question conceptually related to identifiability problems in dynamical systems theory (Bellman and Åström, 1970): when we observe multimodal signals, are we measuring the same dynamics viewed in different ways, or rather different dynamics in the same way?

Numerous latent-variable models have been proposed for analyzing neural data, including nonlinear state-space models, hidden Markov models, and deep generative approaches (Pandarinath et al., 2018; Sussillo et al., 2016; Yu et al., 2008). While powerful, such models often involve high-dimensional latent spaces and observation functions that are less interpretable. In contrast, our approach emphasizes interpretability by explicitly modeling system- versus observer-level contributions in low-dimensional generative frameworks.

Specifically, we introduce a framework that can be used to solve this ambiguity by leveraging the addition of noise, which reveals additional terms that depend only on the properties of the observer, and not of the system. These noise-induced terms can be used as diagnostic tools to determine whether multimodal time series reflect differences in neural dynamics or in observer functions. This strategy aligns with a broader movement in systems neuroscience toward integrative, cross-scale inference (Breakspear, 2017), and theoretical proposals that advocate hierarchical generative models for capturing multiscale neural dynamics (Friston, 2008; Marreiros et al., 2009).

We lay out this work in three main stages: First, we build the theoretical foundation by demonstrating how Stratonovich calculus helps to break system-observer ambiguity. Second, we validate this approach by showing that we can successfully distinguish ground-truth synthetic timeseries generated using forward models. Third, we apply the framework to simultaneous recordings from microwires and macroelectrodes in the human hippocampus.

## 2 Materials and methods

In this section, we outline our approach to disambiguating system- and observer-dependent differences in multimodal recordings. We first describe the nature of the problem and a simple generative model that accommodates the key differences in model structure. We describe how this model was used to both generate synthetic data and explain empirical data by using Bayesian model comparision in assessing observer vs. system-dependent differences. Finally, we introduce a stochastic state-space model–using a Stratonovich formulation of random fluctuations–under which the model comparison procedures were reapplied. This allows us to quantify the extent to which noise assists in separating system and observer effects.

### 2.1 System/observer degeneracy

**FIGURE 1:**
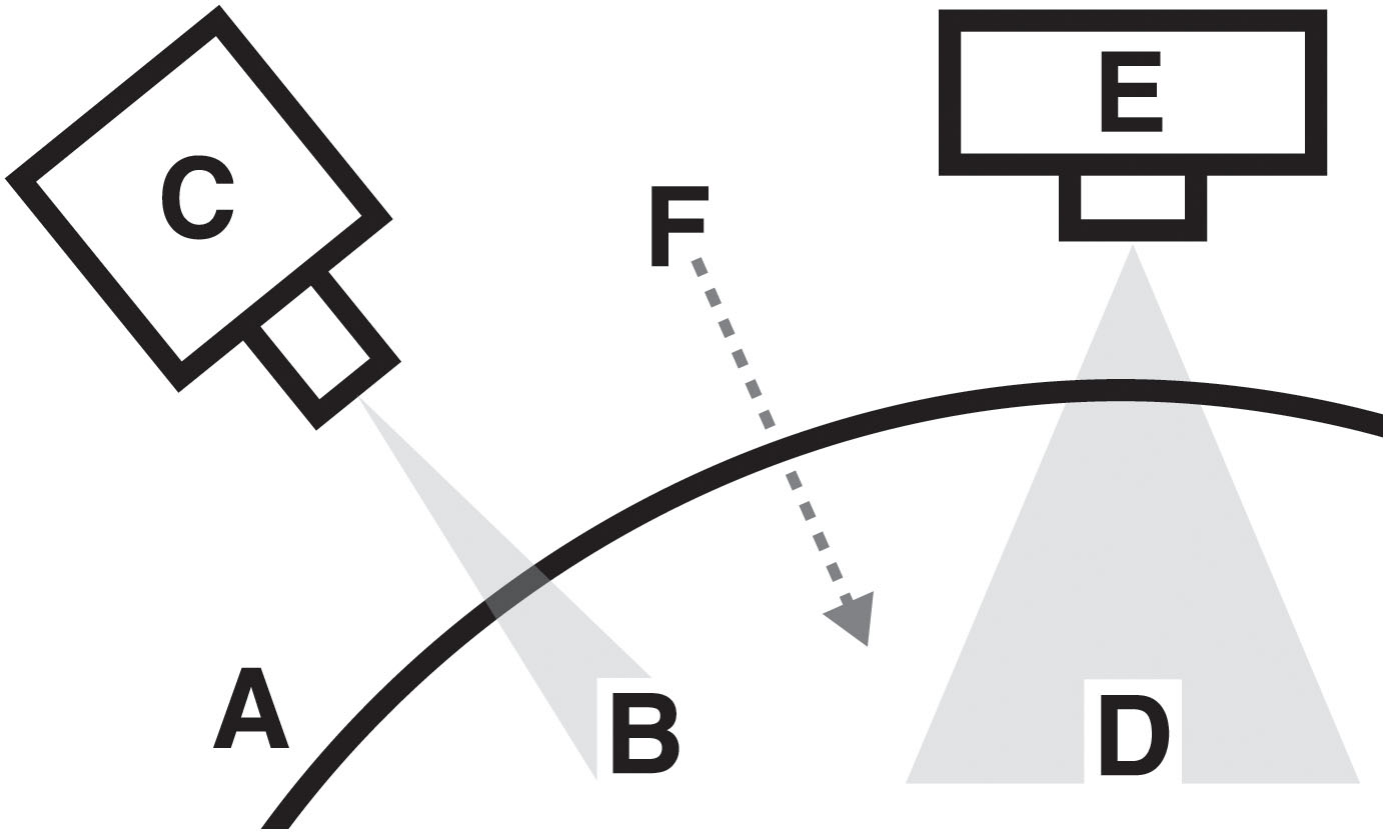
**(A)** The surface of a neural system. **(B)** Small spatial region within the system. **(C)** Neuro-imaging device recording states from region **(B)**. **(D)** Larger spatial region within the same system. **(E)** Second device recording states from region **(D)**. **(F)** External input (dashed arrow) driving activity in both regions.

Consider a neural system (Figure 1A) observed simultaneously by two different imaging devices. A small spatial region (Figure 1B) generates neural signals recorded by one device (Figure 1C), while a larger region (Figure 1D) generates signals recorded by another device (Figure 1E), with each device operating at a different spatial and temporal resolution. Both regions are driven by a common external input (Figure 1F), such as a stimulus or experimental condition that modulates activity across scales.

We model the small-scale region with microscopic state variables *x_μ_* (*t*) that evolve under a function *f_μ_* (*x_μ_*, υ), where υ (*t*) is an exogenous input. The larger region is described by macroscopic states *x*_*M*_ (*t*) evolving under *f*_*M*_ (*x*_*M*_, υ). This gives:


(1) $${\dot{x}}_{\mu }=f_{\mu }\left(x_{\mu },\upsilon \right)\;,{\dot{x}}_{M}=f_{M}\left(x_{M},\upsilon \right).$$

Each device applies an observer function to convert latent neural activity into a measurable signal. The small-scale device uses *g_μ_* (*x*) to produce a signal *h_μ_* (*t*), while the large-scale device uses *g*_*M*_ (*x*) to produce *h*_*M*_ (*t*). In other words, each device applies its own observer function to convert latent neural states into a measurable signal:


(2) $$h_{\mu }\left(t\right)=g_{\mu }\left[x_{\mu }\left(t\right)\right]\;,h_{M}\left(t\right)=g_{M}\left[x_{M}\left(t\right)\right].$$

Taking time derivatives of Equations 1, 2 and applying the chain rule, we obtain:


(3) h.μ=gμ(xμ)′fμ(xμ,υ)  ,h.M=gM(xM)′fM(xM,υ).

Omitting dependent variables for clarity, the difference △ between the observed time derivatives Equation 3 becomes:


(4) △=h.μ-h.M=gμ⁢fμ′-gM⁢fM′

This expression reveals an ambiguity: without additional constraints we cannot know whether differences in measured activity across scales arise more from differences in the underlying system dynamics (*f_μ_*, *f*_*M*_), as opposed to differences in the measurement process itself (*g_μ_*, *g*_*M*_). This ambiguity arises because the difference △ in Equation 4 is expressed in terms of a product between the observer sensitivity and the system dynamics, hence creating a degeneracy – differences cannot be uniquely attributed to either system or observer without further constraints. This is a key motivation for introducing structured noise in later sections, as it enables these multiplicative components to be statistically disentangled.

### 2.2 Generative model

To explore system-observer degeneracy in practice, we define a simple generative model, in which the dynamics of each region are governed by a linear time-invariant (LTI) (Oberst et al., 2020) system:


(5) $${\dot{x}}_{\mu }=ax_{\mu }+b\upsilon \;,{\dot{x}}_{M}=\left(a+\delta a\right)x_{M}+b\upsilon ,$$

Here, *a* governs intrinsic dynamics (Hespanha, 2018), *b* the stimulus gain (Salinias and Sejnowski, 2001), and δ*a* encodes deviations in the macroscopic dynamics system.

Note that, although real neural dynamics are nonlinear, we model both the micro- and macro-scale latent dynamics in Equation 5 using linear approximations. We do this to simplify the process of isolating system- versus observer-level effects. However, we ensure that the generative model allows for nonlinearities via sigmoidal observer functions:


(6) $$g_{\mu }=c\cdot tanh[kx_{\mu }]\;,g_{M}=c\cdot tanh[\left(k+\delta k\right)x_{M}],$$

Here, *c* defines the degree of nonlinearity; *k* sets the input gain, and δ*k* represents macroscopic deviations in the observation process.

### 2.3 Identical observer/system edge cases

Based on the generative model in Equations 5, 6, we now consider two reduced models:

Reduced model 1 – identical observers: To model pure system-level differences, we fix the observation mappings (δ*k* = 0) so that all variation arises from dynamics:


(7) $${\dot{x}}_{\mu }=ax_{\mu }+b\upsilon \;,g_{\mu }=c\cdot tanh(kx_{\mu }),$$

$${\dot{x}}_{M}=\left(a+\delta a\right)x_{M}+b\upsilon \;,g_{M}=c\cdot tanh(kx_{M}),$$

Reduced model 2 – identical systems: To model pure observer-level differences, we fix the dynamics (δ*a* = 0) and allow the observation mappings to vary:


(8) $${\dot{x}}_{\mu }=ax_{\mu }+b\upsilon \;,g_{\mu }=c\cdot tanh(kx_{\mu }),$$

$${\dot{x}}_{M}=ax_{M}+b\upsilon \;,g_{M}=c\cdot tanh(\left(k+\delta k\right)x_{M}),$$

We then generate synthetic time series from each reduced model in Equations 7, 8. These serve as ground truth datasets for Bayesian model inversion, where we ask whether one can correctly infer if observed differences originate from system or observer-level variation.

### 2.4 Breaking system/observer degeneracy with noise

We now introduce the central hypothesis of this paper: that adding stochastic input (i.e., state or system noise) to the model can improve model identifiability. To illustrate this, let us return to the equations of motion for the micro and macroscopic states *x_μ_* and *x*_*M*_ from Equation 1 and augment them with stochastic inputs:


(9) $${\dot{x}}_{\mu }=f_{\mu }+\sigma_{\mu }dW\;,{\dot{x}}_{M}=f_{M}+\sigma_{M}dW$$

where σ_μ_,σ_*M*_ are volatility constants, and *dW* an increment of a Wiener process. This transforms the generative model from an ordinary differential equation (ODE) form into the stochastic differential equation (SDE) form in Equation 9.

Following Equations 3, 4 [but now using Stratonovich calculus (Mauri and Mauri, 2013)], the difference △_*Strat*_ between observed time derivatives becomes:


(10) $$\Delta_{Strat}=\underset{degenerate}{\underset{︸}{g_{\mu }{}^{\prime }f_{\mu }-g_{M}{}^{\prime }f_{M}}}+\underset{Observers\;only}{\underset{︸}{\sigma_{\mu }\xi_{\mu }g_{\mu }{}^{\prime }-\sigma_{M}\xi_{M}g_{M}{}^{\prime }}}$$

Here, $\xi \,\left(t\right)=\dot{W}$ is formally read as a white noise process.

This highlights the same ambiguity described earlier in Equation 4, where differences in observed activity may arise from either system or observer effects.

We see from Equation 10 that the first difference (i.e., between the first two terms on the right-hand side of the equation) reflects the degenerate overlap between system and observer effects, while the second difference is attributable exclusively to observer sensitivity to noise. These pure observer-based terms arise from differential responses of the observer functions to the same stochastic input. Crucially, these observer-only terms break the degeneracy present in the noise-free model by introducing variability that is independent of the system dynamics *f_μ_*, *f*_*M*_.

To empirically test whether state noise can help in disambiguating system vs. observer effects, we apply this stochastic augmentation to the deterministic model used in Equation 5:


(11) $${\dot{x}}_{\mu }=ax_{\mu }+b\upsilon +\sigma_{\mu }\xi \;,{\dot{x}}_{M}=\left(a+\delta a\right)x_{M}+b\upsilon +\sigma_{M}\xi ,$$

In all models, including the deterministic and stochastic variants, observation noise was also included as an additive term on the observer output, capturing variability in the measurement process.

### 2.5 Empirical data

We analyzed intracranial recordings from 16 human participants (Keles et al., 2024), acquired using Behnke-Fried hybrid depth electrodes (Carlson et al., 2018) while participants passively viewed an 8-min movie excerpt. Each electrode contained 8 microwires (Einevoll et al., 2013) and 8 macroelectrodes (Lachaux et al., 2003), recording broadband field potentials at 32 kHz and 2 kHz, respectively. From these recordings, we extracted HFB power in the 70–170 Hz range, which we refer to throughout as micro-HFB (from microwires) and macro-HFB (from macroelectrodes). A shared movie stimulus clock was synchronized across systems using TTL pulses to ensure precise temporal alignment of neural signals with the audiovisual stimulus.

Data were pre-processed using a standard pipeline: notch filtering at 60 Hz and harmonics to remove line noise, high-pass filtering at 0.1 Hz to remove slow drifts, and re-referencing to the common average. Time-frequency decomposition was performed using Morlet wavelets (Cohen, 2019) across the 70–170 Hz range, and the resulting high-frequency broadband (HFB) power was averaged across frequencies to produce a single HFB time course per channel.

We analyzed the simultaneously acquired micro- and macro-HFB signals, averaged within the right hippocampus, downsampled from 1 kHz to 250 Hz and z-scored independently. Each time series was truncated to a common 4-s window (1000 time points) to ensure equal length across modalities. To model exogenous input, we constructed a design matrix encoding scene structure using annotated movie cuts: each scene was assigned a unique value, producing a piecewise constant signal that tracked stimulus transitions. The subjects’ datasets are shown in Supplementary Figure 1.

### 2.6 Synthetic data

We generated synthetic data that mimicked the empirical HFB signal profile by tuning model parameters to approximate the empirical signal statistics. This involved first constructing an exogenous input to replicate stimulus structure, and second fitting model dynamics to the empirical signal.

**FIGURE 2:**
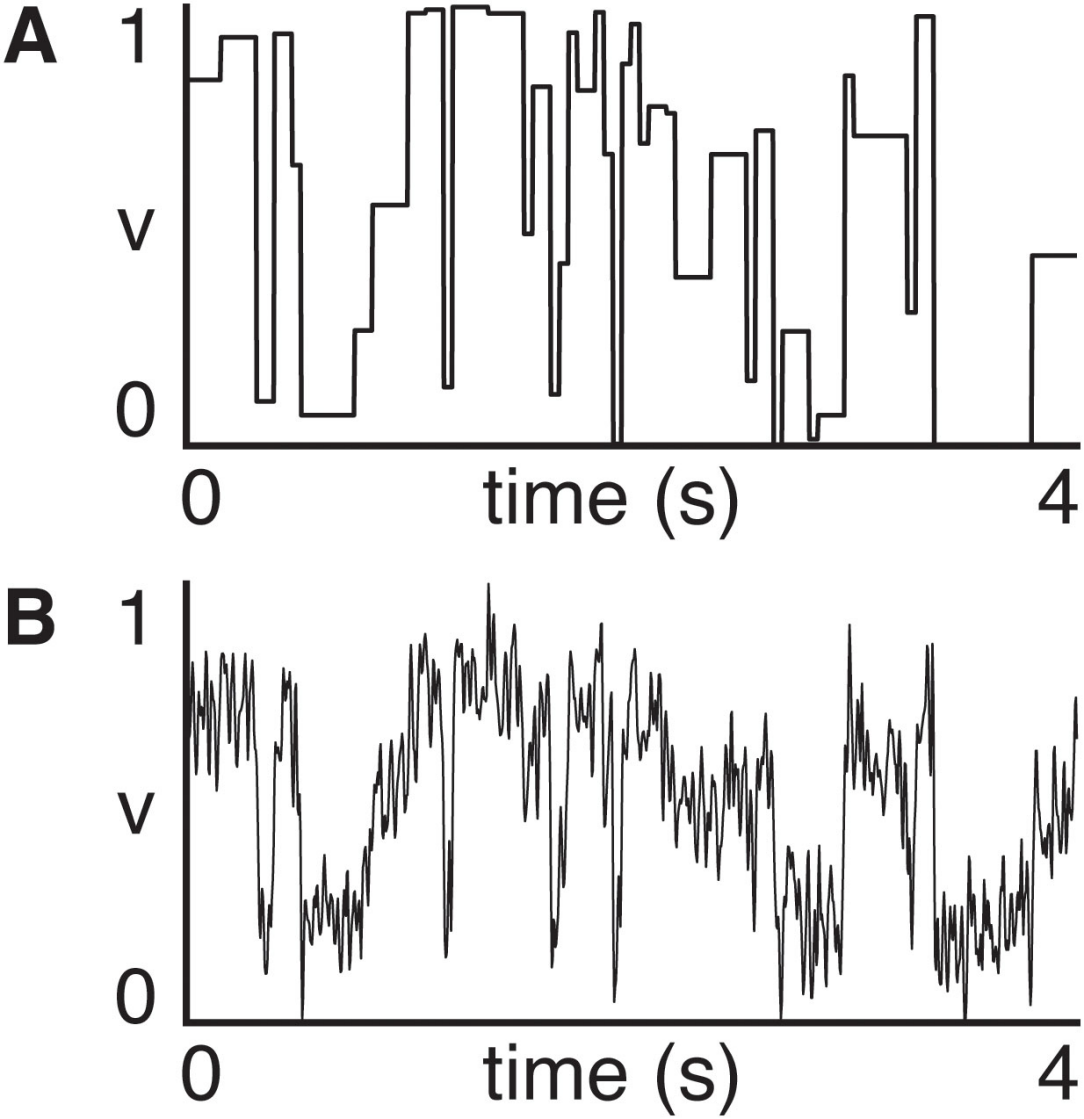
**(A)** Scene-based input signal (v, arbitrary units) constructed by assigning a random value to each annotated scene segment. **(B)** Final driver used in simulations, obtained by adding Gaussian noise and smoothing the signal in panel **(A)**, introducing fast fluctuations alongside the structured scene transitions.

The exogenous input υ (*t*) was constructed to emulate the structure of the movie stimulus by assigning a constant value to each scene. We used annotated scene cut times from the original experiment and defined a piecewise constant signal in which each scene was assigned a random value between 0 and 1 (Figure 2A). To better approximate the complexity of real neural input, we then added Gaussian noise and applied light temporal smoothing, yielding a final driver signal with both slow transitions and fast fluctuations (Figure 2B). This driver served as the shared input to both the microscopic and macroscopic systems in all simulations.

To generate synthetic data resembling empirical HFB activity, we performed a grid search over key model parameters: the intrinsic dynamics *a*, observer nonlinearity *c*, and input gain *k*. For each parameter combination, we simulated time series using the forward model and compared the resulting synthetic output to empirical data (truncated to a 4-s window) using mean squared error. This procedure yielded synthetic time series that preserved the qualitative features of the empirical recordings while retaining explainability under a known generative model.

### 2.7 Bayesian model inversion

We begin with the full model in Equations 5, 6 with two free parameters δ*a* and δ*k*. We assigned standard normal priors (mean zero, unit variance) to both parameters and used Dynamic Causal modeling (DCM) (Friston et al., 2003) to invert the model, with the ground truth datasets generated from the two reduced models in Equations 7, 8. This inversion yields estimates of the posterior over δ*a* and δ*k* for each dataset.

We then apply post-hoc Bayesian model reduction (Friston and Penny, 2011) to determine which reduced model (identical observers vs. identical systems) best explains the data. Specifically, to test for observer-level differences, we fix the δ*a* parameter to zero by setting both its prior expectation and variance to zero, i.e., *Var* (δ*a*) = 0. This evaluates whether a model with identical system dynamics can explain the data. Similarly, to test for system-level differences, we collapse the prior variance over observer deviations: *Var* (δ*k*) = 0. This now evaluates whether a model with identical observer mappings can explain the data.

The log of the model evidence (a.k.a., marginal likelihood) was approximated using the variational free energy (VFE) (Friston, 2010) (a.k.a., evidence lower bound), which quantifies the balance between accuracy and complexity. The preferred model is the one with the higher VFE following model inversion and reduction. This allows us to determine whether Bayesian model comparison can correctly associate each reduced model with its known generative process. Once this proof of principle has been established, we then apply the same technique to each subject’s empirical micro/macro HFB recordings, to determine whether observer- or system-level effects are more prevalent in each.

See the Supplementary Appendix for details on the DCM used.

### 2.8 Multiplicative noise extension

As a supplementary analysis, we test whether the disambiguation effect observed with additive noise also holds when using multiplicative (state-dependent) noise. This involves replacing the additive terms *σ_μ_ξ* and *σ_M_ξ* in Equation 11 with multiplicative terms:


(12) $${\dot{x}}_{\mu }=a\,x_{\mu }+b\,\upsilon +\sigma_{\mu }\,x_{\mu }\,\xi ,{\dot{x}}_{M}=\left(a+\delta \,a\right)\,x_{M}+b\,\upsilon +\sigma_{M}\,x_{M}\,\xi ,$$

This formulation increases the variance of fluctuations with state magnitude, consistent with empirical findings in motor control and neurodynamics (Faisal et al., 2008). Synthetic time series were generated using the model in Equation 12, and Bayesian model inversion and reduction were performed as with the previous additive noise data.

### 2.9 Model fit evaluation

To assess model fit quality, we computed the coefficient of determination *R*^2^ between the predicted and empirical macro-HFB, as well as two residual diagnostics: skewness and autocorrelation at lag 1 (AC1). *R*^2^ values quantify explained variance, with negative values indicating worse-than-baseline fits. Skewness was included to detect non-Gaussian residuals that might indicate unmodeled noise structure, and autocorrelation was used to identify temporal dependencies in the residuals. All metrics were computed after z-scoring the predicted and empirical signals, and residuals were computed as their difference. Subjects were excluded if *R*^2^ < 0.1 and/or if |*skewness*| > 3.

## 3 Results

All results below can be reproduced using the accompanying code.

### 3.1 Synthetic data

**FIGURE 3:**
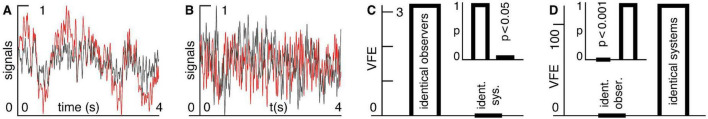
**(A)** Normalized signal strength as a function of time for synthetic data, with the small-scale system shown in black and the large-scale system shown in red. The forward generative model constrains the observer functions to be identical, so that all variation between the two signals is due to differences between systems (i.e., between equations of motion). **(B)** Same as panel **(A)**, except now the systems are identical, and all variation between the two signals is due to differences between observer functions. **(C)** Variational free energy (VFE) for the two reduced models in which (1) observers and (2) systems are identical. VFE values are plotted relative to the minimum within each comparison, such that the lower evidence model is set to zero. In this case, the model selection correctly identifies the “identical observers” reduced model as the one responsible for the generation of the timeseries in panel **(A).** The inset shows probabilities derived from the VFE, showing a significance level of *p* < 0.05. **(D)** Same as panel **(C),** except here the model selection is applied to the timeseries in B), which is correctly identified as the reduced model in which systems are constrained to be identical. The probabilities shown in the inset indicate a high significance of *p* < 0.001.

We begin by creating two synthetic datasets–one with identical observers as in Equation 7 (Figure 3A), and the second with identical systems as in Equation 8 (Figure 3B). We then perform Bayesian model inversion on these two synthetic datasets by employing the full model in Equations 5, 6. We find that both the identical observers (Figure 3C), as well as the identical systems (Figure 3D) ground truth datasets can be correctly identified using Bayesian model reduction.

**FIGURE 4:**
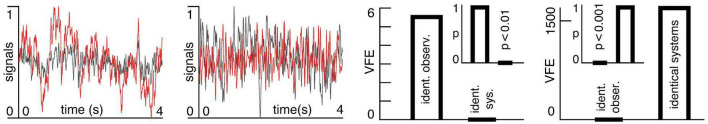
Same layout as Figure 3, except both forward generative models and model inversions now include stochastic terms.

We then repeat the same process, but this time the identical observers (Figure 4A) and identical systems (Figure 4B) data are generated with a white noise driving input. Preserving this stochastic element in the model inversion process, we find that the inclusion of noise facilitates improved disambiguation by increasing the difference between variational free energy (model evidence) for both the identical observers (Figure 4C) and identical systems (Figure 4D) timeseries.

Synthetic tests with multiplicative noise (Supplementary Figure 2) showed similar disambiguation between system and observer effects. This suggests that the framework is robust to different noise structures and not contingent on linearity or additive noise assumptions.

This synthetic data analysis validates the premise that the inclusion of state noise in the models help in breaking system-observer degeneracy. Having established the face validity of this Bayesian model comparison procedure, we can now apply it to empirical data and ask whether micro and macro HFB signals are best explained by observation or system level differences.

### 3.2 Empirical data

**FIGURE 5:**
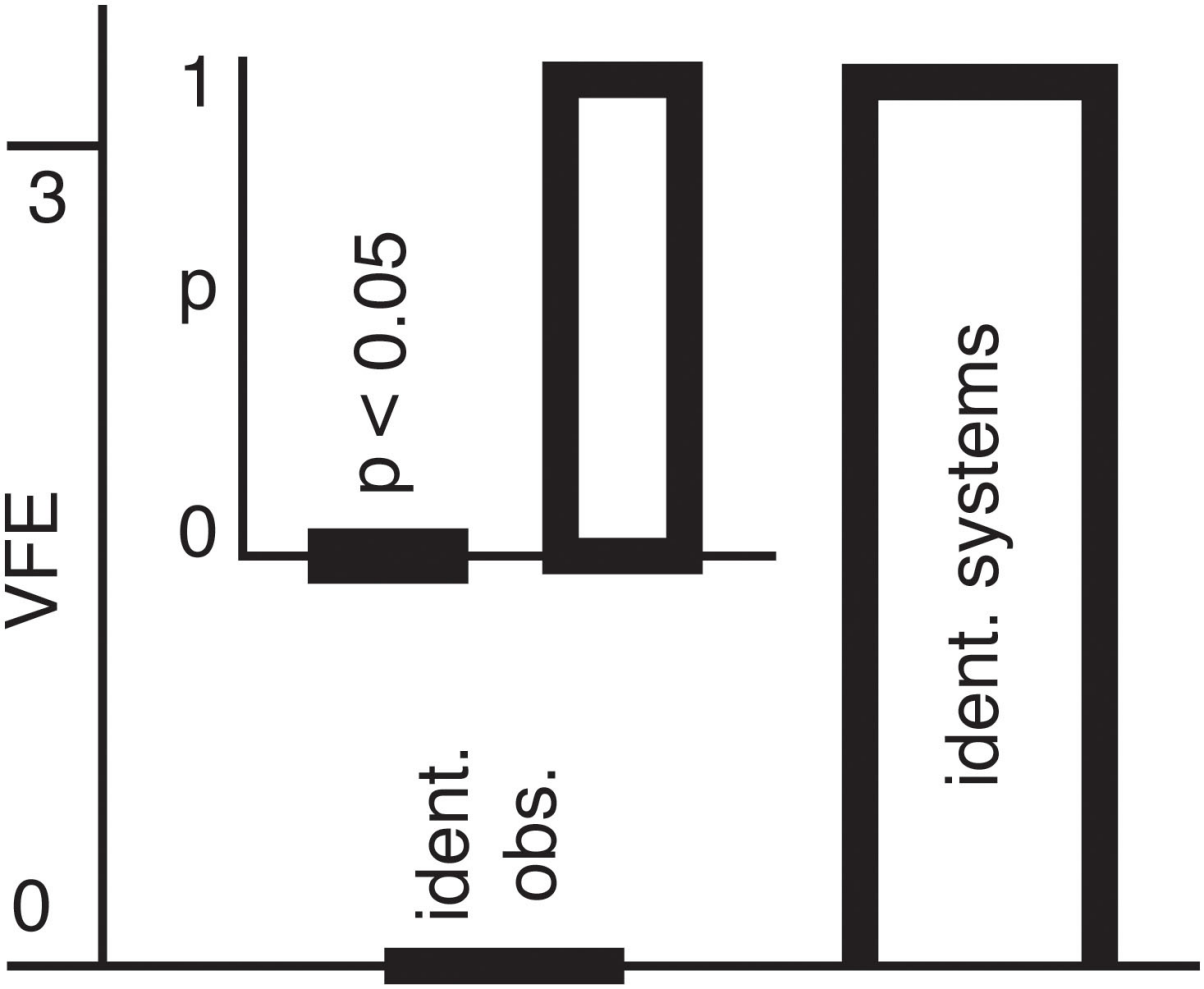
The variational free energy (VFE) is shown on the vertical axis for the two models with (1) identical observers and (2) identical systems for the one subject (ID 2) surviving statistical correction. VFE values are plotted relative to the subject-level minimum, such that the lower bound is set to zero. The inset shows probabilities derived from the VFEs. The “identical systems” model has higher evidence.

Model fit diagnostics were calculated for all 16 subjects (Supplementary Figure 3). Overall, the model accounted for a substantial portion of variance in most subjects (median R^2^ is 0.66), with low residual skewness (median skewness 0.02) and moderate autocorrelation (median AC1 is 0.52). However, four subjects (IDs 4, 7, 13, 16) exhibit negative R^2^ values and/or absolute value skewness larger than 3, suggesting the model failed to capture meaningful structure in these subjects. Upon applying model inversion and reduction to the remaining 12 subjects, one (subject ID 2) presents significantly higher evidence for the “identical systems” compared with the “identical observers” hypothesis. This suggests that the variation between the micro- and macro-HFB recordings in this subject are driven by differences in the observer functions, rather than by the equations of motion governing the two scales (Figure 5).

We show complementary results with multiplicative, instead of additive, noise in Supplementary Figure 4. This shows that the data pertaining to the single subject surviving statistical correction (ID 11) is also driven by differences in the observer function.

## 4 Discussion

This study demonstrates that the inclusion of noise in generative models of neural activity can act as a diagnostic tool, allowing for improved disambiguation between system- vs. observer-induced variation between cross-scale neuroimaging datasets. This disambiguation occurs when nonlinear observer functions are combined with stochastic system dynamics, which results in interaction terms that introduce dependencies specific to the observer only (i.e., and not to the system).

Using synthetic data, we validated this framework by generating ground truth time series under conditions where the systems differed but the observers were identical, and vice versa. In both cases, Bayesian model inversion recovered the correct underlying model more accurately when stochastic inputs were included. This finding formalizes the core point addressed here: system and observer effects are degenerate in the absence of structured noise. As such, without stochastic perturbations passing through nonlinear mappings, the two sources of variation remain less statistically distinguishable. While it might seem intuitive that stochastic models better explain stochastic data, our results go beyond improved fit: they show that noise interacts with nonlinear observer functions in a way that amplifies otherwise latent distinctions between system- and observer-level structure. In this sense, noise is not simply a source of variance, but a probe that reveals model-specific dependencies.

We extended our synthetic tests to include multiplicative noise, in which the amplitude of fluctuations scales with the magnitude of the state variable. The results confirm that our key conclusion – that noise improves disambiguation between system and observer contributions – remains valid. This supports the generality of the approach beyond specific assumptions of linearity or constant volatility.

We then applied this approach to empirical data from 16 human subjects with simultaneous micro- and macro-HFB recordings from the hippocampus. The results indicate a complicated mixture of system and observer effects in all subjects except one – which demonstrated statistically significant evidence of observer-based variation. A different single subject showed the same result when using an assumption of multiplicative, rather than additive, noise. This finding suggests the possibility that micro- and macro-HFB are in some cases observing the same underlying neural processes, just with different observer functions (Buzsáki et al., 2012; Kajikawa and Schroeder, 2011). It should be stressed however, that our method does not assume that such a distinction will always emerge, but rather that detection is enabled when such a distinction is present.

This interpretation aligns with previous work suggesting that micro- and macro-HFB, while often treated as separate levels of analysis, may tap into overlapping neural generators (Mukamel and Fried, 2012), particularly when recorded from the same anatomical region. More speculatively, the results may reflect a broader principle in neuroimaging: that device-level transformations can dominate the differences seen across modalities, especially when those devices are sampling from overlapping regions (Daunizeau et al., 2009). In such cases, modeling those observer functions explicitly–and leveraging noise to expose their structure–may be more fruitful than assuming fundamentally distinct neural sources.

Micro- and macro-HFB signals both originate from spatiotemporally synchronized synaptic potentials, reflecting the evolution of neural activity at different spatial scales (Buzsáki et al., 2012). These synaptic potentials generate approximately dipolar fields, for which the observed polarity depends on electrode placement. Similar recordings in macaque visual cortex showed that the spatial spread of iEEG (∼3 mm) is roughly three times that of LFP, and that iEEG can be modeled as a spatial average of LFP signals over this ∼3 mm radius (Ray, 2023). Consequently, iEEG and LFP are expected to capture similar underlying synaptic dynamics, and differences between their signals can be largely attributed to differences in electrode locations.

It should be noted that the generative models presented do not aim to explicitly model the physical characteristics of electrodes or recording pipelines. Rather, the observer functions serve as mathematical stand-ins for the fact that different devices impose distinct, often nonlinear, transformations on the underlying signals. Our results therefore do not provide a literal mapping between device and model, but rather illustrate a more general principle: that structured noise serves to assist in uncovering the relative contributions of system and observer. In this sense, our findings complement prior work on the role of precision in the identifiability of model components, particularly within the context of dynamic causal modeling (Daunizeau et al., 2009). This builds on prior theoretical work suggesting that noise can enhance inference by breaking model symmetries (Seung and Sompolinsky, 1993).

It should be noted that our method relies upon an appropriately chosen observer function for a given dataset. Otherwise, incorrect assumptions about e.g., nonlinearities could lead to potential confounds that mimic system-level effects. This underscores the need for careful model selection procedures (Daunizeau et al., 2011; Friston, 2009; Razi et al., 2015).

Future implementations of our methodology could involve more complex scenarios, such as the simultaneous acquisition of EEG and fMRI data. The neural mechanisms at the microscopic level that give rise to fMRI BOLD signals remain an open question (Ekstrom, 2010). EEG captures spatiotemporally synchronized synaptic inputs on the millisecond timescale, whereas fMRI measures local changes in blood flow occurring over several seconds. Accurate interpretation of these signals would require more sophisticated modeling of the corresponding observer functions – biologically realistic models for EEG to account for volume conduction, and hemodynamic models for fMRI.

As neuroimaging datasets increasingly span spatial and temporal scales–from cellular-resolution imaging to whole-brain recordings–the challenge of comparing signals across modalities becomes more pressing. Our results suggest that, rather than treating noise as an element, to be eliminated, it can be exploited as a structured source of information–as a diagnostic tool for resolving ambiguity in multimodal signals.

## Data Availability

Publicly available datasets were analyzed in this study. This data can be found here: https://github.com/rutishauserlab/bmovie-release-NWB-BIDS. All MATLAB code used to produce results is made available at: https://github.com/allavailablepubliccode/system_v_observer.
