# Supplementary material for: Using noise to distinguish between system and observer effects in multimodal neuroimaging
Source: Front Comput Neurosci. 2025 Oct 17;19:1693279. doi: 10.3389/fncom.2025.1693279 (PMC12575338; doi:10.3389/fncom.2025.1693279)
Supplement: Supplementary file 1 [file Data_Sheet_1.docx]

**Supplementary Figure 1:** Normalized signal (sig.) strengths (arbitrary units) over time (0–1000 samples) per subject, running from top left to right and top to bottom. Black and red traces indicate micro- and macro-HFB signals, respectively. All panels share the same axis limits (see top left).

**Supplementary Figure 2:** Same layout as Fig. 4, except for multiplicative instead of additive noise.

**Supplementary Figure 3:** Red outlines indicate subjects excluded due to poor fit. Metrics include $R^{2}$ of predicted vs. empirical signal (top), skewness of residuals (middle), and residual autocorrelation at lag 1 (bottom).

**Supplementary Figure 4:** Same layout as Fig. 5 showing the one subject (ID 11) surviving statistical correction in the case of multiplicative noise.

**Appendix**

Model Inversion and Bayesian Comparison using DCM Model Overview: To assess system vs. observer contributions to neurophysiological signals, we implemented a generative model in the Dynamic Causal Modelling (DCM) framework using the Laplace approximation (LAP) as implemented in SPM12 (Karl Friston, Stephan, Li, & Daunizeau, 2010).

Each subject’s data was modelled as a simple two-state dynamical system, comprising a system and a 'twin' that differ only by a single parameter (either in the system dynamics or the observer function). Both states are driven by shared external inputs, allowing differences in model evidence to be attributed to structural asymmetries.

Model Equations: Let $x\left( t \right)$ and $x_{t}\left( t \right)$ denote the latent state variables of the system and its twin. The equations of motion are:

$$\dot{x}=ax+bu_{1}+s_{1}u_{2}$$

$$\dot{x}_{t}=\left( a+\delta a \right)x_{t}+bu_{1}+s_{2}u_{2}$$

where $u_{1}\left( t \right)$ is a structured scene-based regressor and $u_{2}\left( t \right)$ is fast Gaussian noise. The observation function maps these latent states to observable quantities via:

$$y=c\cdot tanh\left[ kx \right]$$

$$y_{t}=c\cdot tanh\left[ \left( k+\delta k \right)x_{t} \right]$$

In this formulation, differences in dynamics are introduced by $\delta a$, while differences in observation are captured by $\delta k$.

All parameters had zero prior expectations (i.e., null hypothesis of identity between system and twin). The prior covariances were soft identity matrices scaled by a variance factor (here, 0.5) to allow some flexibility without overwhelming the empirical data.

Data and Inputs: Each subject’s data consisted of paired macro- and micro-electrode high-frequency broadband (HFB) signals recorded from the right hippocampus. These signals were downsampled to 250 Hz and z-scored before modelling. The external input $u_{1}\left( t \right)$ was derived from scene-change timestamps, simulating structured cognitive or perceptual fluctuations, while $u_{2}\left( t \right)\mathcal{\sim N}\left( 0,1 \right)$ was synthetic noise.

Model Inversion: Models were inverted using spm_LAP, which implements dynamic expectation maximization under a Laplace approximation. The embedding dimension was set to $E.n=4$ and smoothness to $E.s=1/8$. We used 8 Newton iterations per sample.

The model inverts two coupled latent processes given observed macro and micro LFP signals. Hyperpriors on noise precisions (input and state noise) were automatically inferred via spm_LAP_eval, which evaluates the precision functions over time given the latent trajectories. This allows the model to estimate time-varying precision of both hidden states and inputs.

Bayesian Model Reduction and Evidence Comparison: After inversion, each subject’s full model posterior was reduced using spm_dcm_reduce, which re-evaluates the evidence (variational free energy) under restricted priors (e.g., fixing $\delta k=0$ to test identical observers, or $\delta a=0$ for identical systems).

The resulting free energies for each model variant were denoted:

F_1_: Model with difference in observer ($\delta k\neq0 , \delta a=0$)

F_2_: Model with difference in system ($\delta a\neq0 , \delta k=0$)

Softmax-Based Posterior Probability Calculation: Posterior probabilities over models were computed from the (negative) free energy differences using a softmax transform:

$$p_{i}=\frac{e^{F_{i}}}{\sum_{j} e^{F_{j}}}$$

This was implemented using the standard SPM utility spm_softmax. The softmax outputs a categorical distribution over models for each subject, providing a probabilistic estimate of which model best explains the data.

For example, a posterior probability of $p=0.95$ for the identical systems model implies evidence that differences in observation, rather than underlying system dynamics, explain the observed data. Thresholds of $p>0.95$ (i.e., free energy difference $\approx3$) were used to denote statistically credible model preference, following Jeffreys’ scale.

Sensitivity to Parameters: Model comparison in DCM can be sensitive to prior settings, especially prior variance and noise assumptions. For this reason, we deliberately used conservative (soft) priors and flexible noise models to ensure that any detected model preference reflected true dissociable signal structure, not overfitting. While this increased realism, it also raised the bar for detecting systematic differences, resulting in strong evidence for only a single subject in the final model comparison.
