# Supplementary figures and images for: Using noise to distinguish between system and observer effects in multimodal neuroimaging
Source: Front Comput Neurosci. 2025 Oct 17;19:1693279. doi: 10.3389/fncom.2025.1693279 (PMC12575338; doi:10.3389/fncom.2025.1693279)

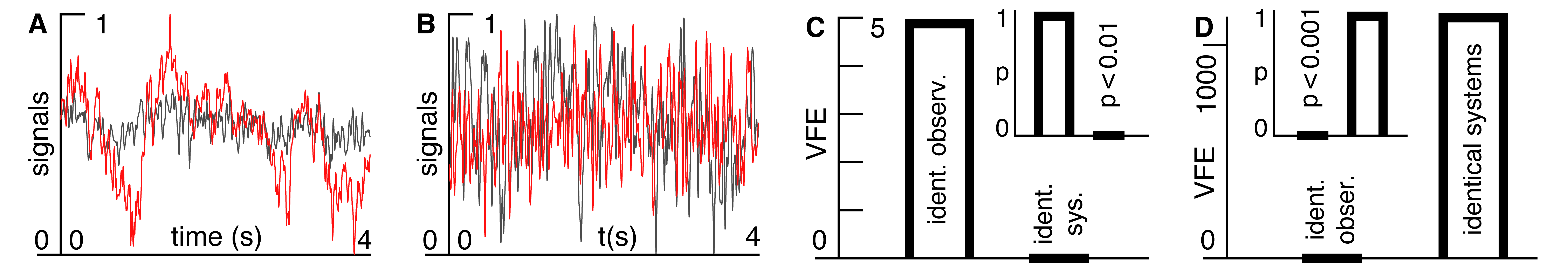

Supplement: Supplementary file 3 [file Image_2.tif]

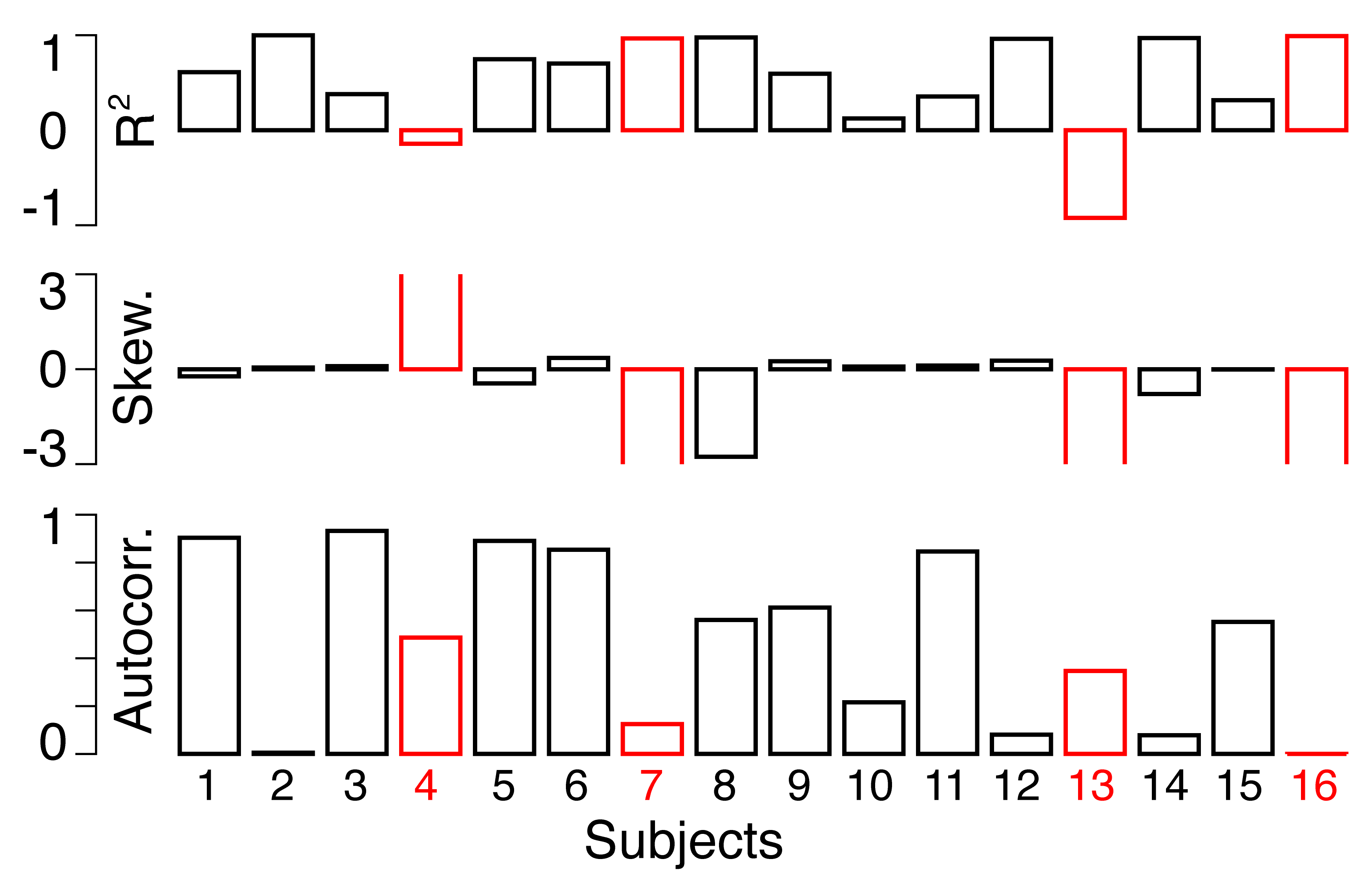

Supplement: Supplementary file 4 [file Image_3.tif]

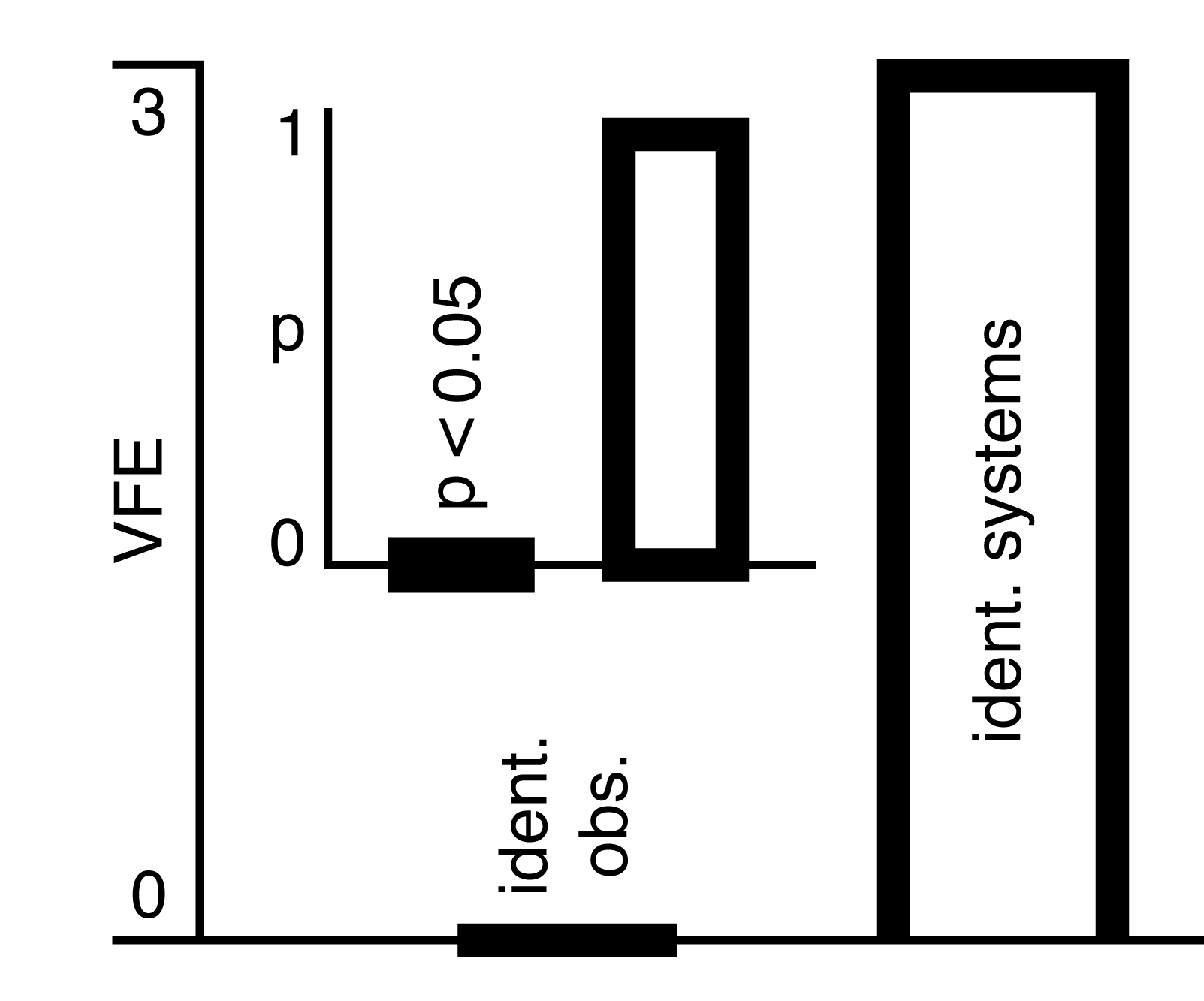

Supplement: Supplementary file 5 [file Image_4.tif]
